# Draft Genome Sequence of a Rare Smut Relative, *Tilletiaria anomala* UBC 951

**DOI:** 10.1128/genomeA.00539-14

**Published:** 2014-06-12

**Authors:** Merje Toome, Alan Kuo, Bernard Henrissat, Anna Lipzen, Andrew Tritt, Yuko Yoshinaga, Matthew Zane, Kerrie Barry, Igor V. Grigoriev, Joseph W. Spatafora, M. Catherine Aime

**Affiliations:** a Department of Botany and Plant Pathology, Purdue University, West Lafayette, Indiana, USA; b U.S. Department of Energy Joint Genome Institute, Walnut Creek, California, USA; c Architecture et Fonction des Macromolécules Biologiques, Centre National de la Recherche Scientifique & Aix-Marseille University, Marseille, France; d Department of Botany and Plant Pathology, Oregon State University, Corvallis, Oregon, USA

## Abstract

The draft genome sequence of the smut fungus *Tilletiaria anomala* UBC 951 (Basidiomycota, Ustilaginomycotina) is presented. The sequenced genome size is 18.7 Mb, consisting of 289 scaffolds and a total of 6,810 predicted genes. This is the first genome sequence published for a fungus in the order Georgefisheriales (Exobasidiomycetes).

## GENOME ANNOUNCEMENT

*Tilletiaria anomala* Bandoni & B. N. Johri is the only species in the fungal genus *Tilletiaria* (1). The only known strain of this species (UBC 951=CBS 436.72=ATCC 24038) was isolated from decayed wood in Canada during an attempt to culture a different fungus (a *Poria* sp.). Originally placed within Ustilaginales (1), *T. anomala* is now known to belong to Georgefisheriales (Exobasidiomycetes) and is related to several *Tilletiopsis* species (2–4). *T. anomala* is dimorphic, producing both hyphal and budding yeast stages in culture. The species is homothallic, and teliospores are intercalary and thick-walled. Because the sole representative of this species is known only from culture, its role in nature is unknown. However, environmental sequencing projects have detected *T. anomala* from the intercellular fluid of rice plants in Japan (5) and from the soil of deciduous forests in Estonia (L. Tedersoo, personal communication), implicating an association with plants, as is true for the majority of Ustilaginomycotina.

Genomic DNA was isolated using the cetyltrimethylammonium bromide (CTAB) protocol described by Padamsee et al. (6), and RNA was extracted with the E.Z.N.A. fungal RNA kit (Omega Bio-Tek, Norcross, GA, USA) following the manufacturer’s instructions. Genome sequencing was accomplished on an Illumina HiSeq 2000 DNA platform according to the manufacturer’s instructions. The genome was assembled using ALLPATHS-LG (7) and annotated using the JGI annotation pipeline (8), following the methods described by Toome et al. (9).

The *T. anomala* genome is contained in 289 contigs of a total size of 18.7 Mb (25× read depth coverage). Annotation of the genome resulted in a set of 6,810 gene models, 93% of which were supported by transcripts that cover >75% of their length. Median gene and protein lengths are 1,718 bp and 407 aa, respectively, gene density is 364 genes Mb^−1^, and the G+C content is 56%. Less than 1% of the genome is repetitive, with virtually no detectable polymorphism. Analyses of the carbohydrate-active enzymes detected 85 enzymes from 34 glycoside hydrolase (GH) families, 47 enzymes from 22 glycosyltransferase (GT) families, and 11 enzymes from two carbohydrate esterase (CE) families. The genome sequence of *T. anomala* provides the first genomic data for a member of the Exobasidiomycetes order Georgefisheriales as well as for a homothallic species of Ustilaginomycotina.

### Nucleotide sequence accession number.

The genome sequences and annotations are available via the JGI fungal genomics resource MycoCosm (10) at http://genome.jgi.doe.gov/Tilletiaria/ and have been deposited at DDBJ/EMBL/GenBank under the accession no. JMSN00000000.
